# PEGylated liposomes enhance the effect of cytotoxic drug: A review

**DOI:** 10.1016/j.heliyon.2023.e13823

**Published:** 2023-02-17

**Authors:** Muhammad Taher, Deny Susanti, Muhammad Salahuddin Haris, Aina Atiqah Rushdan, Riyanto Teguh Widodo, Yandi Syukri, Junaidi Khotib

**Affiliations:** a Department of Pharmaceutical Technology, Kulliyyah of Pharmacy, International Islamic University Malaysia, 25200, Kuantan, Pahang, Malaysia; b Pharmaceutics and Translational Research Group, Kulliyyah of Pharmacy, International Islamic University Malaysia, 25200, Kuantan, Pahang, Malaysia; c Department of Chemistry, Kulliyyah of Science, International Islamic University Malaysia, 25200, Kuantan, Pahang, Malaysia; d Department of Biomedical Science, Kulliyyah of Allied Health Sciences, International Islamic University Malaysia, 25200, Kuantan, Pahang, Malaysia; e Department of Pharmaceutical Technology, Faculty of Pharmacy, University of Malaya, 50603, Kuala Lumpur, Malaysia; f Faculty of Pharmacy, University Islam Indonesia, 55584, Yogyakarta, Indonesia; g Department of Pharmacy Practice, Faculty of Pharmacy, Airlangga University, 60115, Surabaya, Indonesia

**Keywords:** Drug delivery, Cancer, Tumor, Nanoencapsulation, Lipid vehicle, Preparation

## Abstract

Cancer is a second leading disease-causing death worldwide that will continuously grow as much as 70% in the next 20 years. Chemotherapy is still becoming a choice for cancer treatment despite its severity of side effects and low success rate due to ineffective delivery of the chemodrugs. Since it was introduced in 1960, significant progress has been achieved in the use of liposomes in drug delivery. The study aims to review relevant literatures on role of PEGylated liposome in enhancing cytotoxic activity of several agents. A systematic literature on the use of PEGylated liposomes in anticancer research via Scopus, Google scholar and PubMed databases was conducted for studies published from 2000 to 2022. A total of 15 articles were selected and reviewed from 312 articles identified covering a variety of anticancer treatments by using PEGylated liposomes. PEGylated liposome which is purposed to achieve steric equilibrium is one of enhanced strategies to deliver anticancer drugs. It has been shown that some improvement of delivery and protection form a harsh gastric environment of several anticancer drugs when they are formulated in a PEGylated liposome. One of the successful drugs that has been clinically used is Doxil®, followed by some other drugs in the pipeline Various drugs (compounds) had been used to enhance the efficacy of PEGylated liposomes for targeted cancer cells *in vitro* and in vivo. In conclusion, PEGylated liposomes enhance drug activities and have great potential to become efficient anticancer delivery to follow Doxil® in the clinical setting.

## 1 Introduction

Cancer is a major cause of death worldwide resulting in nearly 10 million deaths in 2020, or causing 16% of people's deaths which commonly suffer from breast, lung, colon, rectum and prostate cancer. The treatment approach includes surgery, radiotherapy and chemotherapy [1]. Chemotherapy is widely used in the treatment of many types of cancers using various classes of drugs such as antimetabolite, alkylating agents, natural products, hormones, antagonist and monoclonal antibodies [2].

Various anticancer drugs have been developed and used in clinical settings. However the effectiveness of the anticancer drug that is formulated in a conventional delivery is considered low due to poor solubility and or stability and lack of specificity [3]. One of the strategy to improve these limitations is an improved drug delivery system such as nanocarriers or nano-formulation.

Liposomes are the most successful nanocarriers that have been successfully reaches a commercial level [4]. The name of liposome itself came from the Greek words, “lipos and soma”, meaning fat and body, respectively [5]. Liposomes are globular lipid bilayer with diameter of 50–1000 nm. It is made by phospholipids and cholesterol. To increase its clinical acceptability, liposomes was then developed with polyethylene glycol (PEG) [6].

A nano-formulated PEG-liposomal product which was successfully used in the delivery of doxorubicin known as Doxil® or Caelix® [7,8]. Doxil ® has improved the circulation half-life, slow plasma clearance and reduced volume distribution of doxorubicin compared to the free form [8]. Nano-formulation enhances permeability and retention effect of drug on solid tumor leading 5-folds accumulation compared to the free drug [[7], [8]] . Doxil® requires a single infusion once monthly compared to topotecan which requires daily 30-min for five days every three week in ovarian cancer patients [10]. However, due to unsatisfactory effects of Doxil®with regards to toxicity, then limit the use of Doxil® in the treatment of cancer. To overcome this limitation, some researchers combined doxorubicin with other drugs. Recent studies on other drugs showed that the use of PEGylated liposomes had improved the delivery of some cytotoxic agents [3,11].

## 2 Liposome technology

Liposome technology has become a targeted nanoparticle and drug delivery system as it can produce various formulations to deliver different drugs in preclinical as well as clinical trials. Liposomes are unilamelar or multilamelar vehicles consisting of a phospholipid bilayer typically about 50–450 nm in size [12]. The presence of two aqueous and organic phases in liposome structure allows entrapment of hydrophilic and lipophilic drugs which enhance its specificity, bioavailability and biocompatibility [4].

**Fig. 1:**
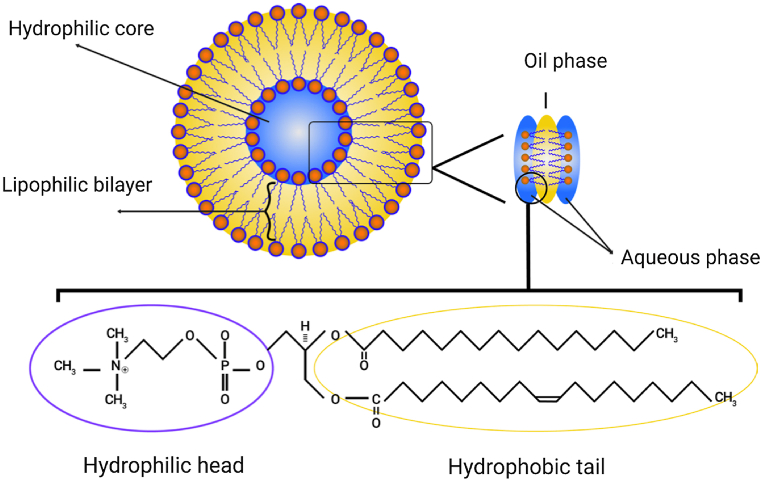
Schematic structure and composition of liposome. Taken from Ref. [4].

The liposome membrane of phospholipids is made from phosphatidylcholine, phosphatidylserine, sphingomyelin and phosphatidylethanolamine which are amphiphilic in nature and have a capability to form particular structure in water [12]. The co-presence of phosphate molecules (hydrophilic head) and fatty acid hydrophobic tails) plays an important role in this physical phenomenon. The phosphate group interacts with water (H_2_O molecules) while the hydrophobic tails escape from water molecules and interact with each other [4]. If non polar chains are put oppositely to each other, it will make a bilayer of lipophilic space in between. This lipophilic part of the liposome can be used to store hydrophobic materials. Then, the hydrophilic part of the phospholipid is facing directly toward the water molecule via hydrogen bond or van der Waals [4]. Natural phospholipid like lecithin is found abundantly in egg yolk and able to form liposomes in water as shown in Fig. 1.

Different strategies have been employed to synthesis liposome. The typical synthesis procedures are using thin layer hydration, mechanical agitation, solvent evaporation, solvent injection and the surfactant solubilization. The liposomes preparation involves three main steps including, vesicle formation, vesicle volume reduction and purification [13]. Liposomes are most commonly prepared by lipid hydration and organic replacement by an aqueous medium [13]. These methods can be conducted either by reverse-phase evaporation or by organic solvent injection. The lipid hydration is followed by manual stirring of by vortex (Bang-ham's method), followed by solvent removal under reduced pressure until a thin film is obtained. Aqueous medium is used to hydrate the film thin film above the phase transition temperature which results in multilamellar vesicle (MLV) liposome formation [13,14]. Then different methods like sonication, extrusion, homogenization or microfuidisation converting MLV to large unilamellar vesicle (LUV) or small unilamellar vesicle (SUV). The purification of liposomes is performed by either centrifugation, dialysis, ultrafiltration or using column chromatography [13].

The effectiveness of the liposome system has been improved by including a polyethylene glycol polymer (PEG)-lipid conjugate into the lipid membrane. This is due to the steric effect caused by the interaction of liposomes with plasma proteins in the blood [15,16]. The steric effect of surface modification of the nano-carrier with polyethylene glycol chains allows it to avoid reticuloendothelial system's absorption. Furthermore, the steric effect can prevent the mononuclear phagocyte system from identifying and phagocytosing drugs [17,18]. Therefore, PEGylated liposomes exhibit positive results such as prolonging drug circulation, enhancing accumulation in tumors, permeability, as well as retention effect and attenuates side effects [19,20].

PEG is formed by a process of linking repeating units of ethylene glycol to form polymers with linear or branched shapes of different molecular masses (Fig. 1). These PEG structures are then chemically attached to the drug of choice in a process called pegylation. PEGylated liposomes have been found to possess higher drug loading capacity up to 90% or more and some drugs like CPX-1 encapsulated in such liposomes have increased the disease control up to 73% of patients suffering from colorectal cancer [13].

## 3 PEGylated technology

Pegylation (PEG) technology was introduced by Frank Davis in the late 1960 to protect enzyme attack and immunogenic reaction of a recombinant protein drug [21]. Nowadays, PEG has been approved by the FDA as a vehicle in many preparations including pharmaceuticals. Incorporation of longer chain PEG reduces the pH responsiveness of PEGylated liposome, although the uptake of cell toward the higher molecules of PEG is low [11]. PEG lacks immunogenic reactions and toxicity. It prolongs circulation time and increases drug stability. PEG structures are chemically attached to the substances in a process called pegylation. PEG has successfully increases potency and pharmacokinetic/pharmacodynamic activities of several drugs such as interferon a2a, interleukin −6 and tumor necrosis factor [3]. [10. PEGylated liposomes have been reported to increase loading capacity up to 90% [13]. PEGylated technology is recognized as a promising drug delivery system since it encapsulates various hydrophilic and hydrophobic active molecules [22,23].

Stealth liposomal drugs show remarkable results, including prolonged circulation half-life and drug clearance reduction [24]. Smaller in structure and size, stealth liposomes help to prevent drug extravasation, and in conjunction with the specific characteristics, the vascular permeability increases as the selective drugs accumulate in the tumor tissues [25,26]. However, the drug concentration in the cardiac muscle remains low. Stealth liposomes also enhance delivery to the central nervous system as they make alterations to the distribution compared to non-nano particles [25]. This is because a prolonged systematic exposure can result in tumor microcirculation permeation through the leaky endothelium by passive convection transport [[27], [28], [29],[37]]. Prolonged half-life circulation has improved the chances of extravasation as well as greater circulation passages through the tumor cells [30].

**Fig. 2:**
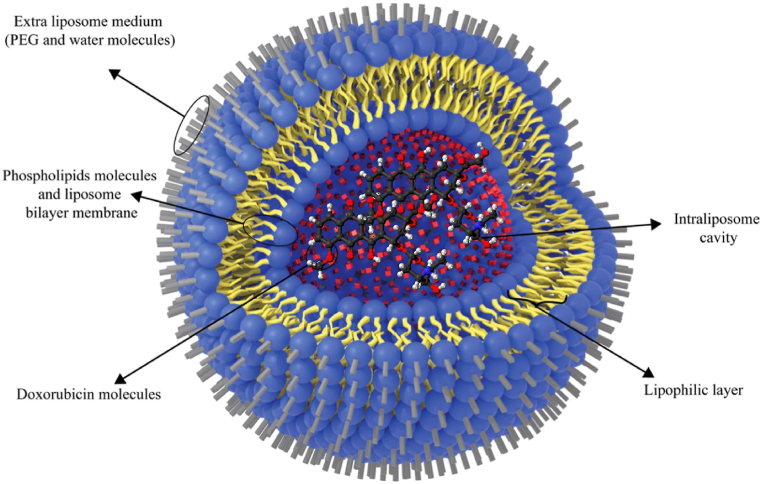
Schematic structure of doxorubicin encapsulated in PEGylated liposome. Doxil® consist of a liquid suspension of unilamellar vesicles with size range from 80 to 90 nm. Taken from Ref. [4].

Polyethylene glycol (PEG) is a significant component in the composition of different nanoparticles because of its stability in the blood circulation, capability to prolong release rates, and high level of water solubility. Antigenicity and immunogenicity are also reduced in PEGylated liposomes [31]. The addition of PEG-lipid conjugates reduces the amount of entrapped chemical that leaks out. As a result, PEGylated liposomes will not only improve drug bioavailability and reduce drug concentration but also provide excellent cancer cell targeting. However, adverse effects remain unavoidable even when a nanoparticulate drug delivery method is used [32] and role of PEG in cell interaction and drug release is still at debate [11,33]. Doxil® consists of a liquid suspension of unilamellar vesicles with size ranges from 80 to 90 nm (Fig. 2). The active ingredient of Doxil® is doxorubicin hydrochloride (C_27_H_29_NO_11_–HCl) with molecular weight of 579.99, cytotoxic anthracycline obtained from *Streptomyces peucetius* var. *caesius*. Total lipid content in Doxil® is approximately 16 mg/mL and doxorubicin content is 2 mg/mL [7].

In this context, the systematic review aims to discover the PEGylated liposome strategies in anticancer drug delivery to increase the residence time of anticancer drugs in the blood circulation, the cytotoxic effects of PEGylated liposomes on cancerous cells to minimize unintended toxicity and enhance therapeutic efficacy and physicochemical properties to improve the drug solubility and stability of PEGylated liposomes including the size, zeta potential, entrapment efficiency (EE), and its effects on cancer cells.

## 4 Methodology

### 4.1 Search strategy and study selection

A systematic review was conducted on the journal articles obtained from online databases to discover the ability of PEGylated liposomes to exhibit longer residence time of anticancer drugs in the blood circulation, investigate the cytotoxic effects on cancerous cells in order to minimize the unintended toxicity and enhance therapeutic efficacy, as well as evaluate the physicochemical properties to improve the drug solubility and stability of PEGylated liposomes. This systematic review was performed using Preferred Reporting Items for Systematic Reviews and Meta-Analyses (PRISMA) method [34]. Population, Intervention, Comparison and Outcome (PICO) was used to guide the establishment of research questions [35]. On the other hand, PRISMA was used to screen and select the articles for the systematic review.

A digital search was applied to find the articles published between January 2000 and September 2022 and was performed using the databases from Scopus, Google scholar and PubMed on the PEGylated liposome strategy for anticancer drug delivery. The articles published between the time range were selected to ensure the comprehensiveness of literature search for PEGylated liposomes on anticancer treatments. The BOOLEAN keywords were also used which included “AND”, “OR” and the characters of double opening and closing quote, as well as the MeSH terms for an effective searching process. The following combination of keywords was used in the search engine, i.e. (“PEGylated liposomes OR PEGylated liposome”) AND (“anticancer OR cancer”) AND (“cancer treatment”) AND (“drug delivery” OR drug”) AND (“anticancer drug”).

### 4.2 Inclusion criteria and exclusion criteria

The inclusion and exclusion criteria are presented in Table 1.

**Table 1:** Inclusion and Exclusion criteria.

Inclusion Criteria	Exclusion Criteria
Studies that used only PEGylated liposomes for anticancer treatments whether in vivo or *in vitro*	Studies that investigated PEGylated liposome delivery on other diseases (unrelated to anticancer treatments)
Studies that were published during 2000–2022	Below 2000
Only original articles	Literature review, commentary, editorial, general information, conference, news, report, and expert opinions
Only English articles with the inclusion of full-text articles	Journals which were duplicates, incomplete, without full-text articles, and published in other languages than English
Studies or articles that critically defined the population, intervention, comparison, and outcomes (PICO) parameters	Non-human and animal studies related to PEGylated liposomes for anticancer treatment use either in-vitro or in-vivo with a low level of evidence

### 4.3 Data extraction

Data from the selected studies were extracted and summarized in Table 2 with regards to types of cells used, substances used, preparation methods, types of pegylation, and effects.

**Table 2:** In-vitro characterization and evaluation tests of the physicochemical properties of some compounds in PEGylated liposomes.

Types of cancer cells	Substances	Methods of preparation	Types of PEGylated liposomes	Effects	References
Human liver hepatocellular carcinoma cell line (HepG2)	Goniodiol	Thin-film hydration method	Goniodiol-loaded PEGylated liposome (PEG-A-DSPE and PEG-P-DSPE)	The amount of goniodiol in PEGylated liposomes was greater than in regular liposomes.	[36]
NT8e oral cancer cell line	5-Fluorouracil (5 FU) and Resveratrol (RES)	Thin-film hydration method	PEGylated dual liposomal formulation	Improved the cytotoxicity in comparison with the free drug	[32]
B16F1 melanoma	Plumbagin (PLB)	Thin-film hydration method	PLB liposomes (PLB Plumbagin)	Enhanced plasma half-life and therapeutic efficacy	[23]
Breast cancer cell line (MCF-7 and T47D)	Anethole [1-methoxy-4-(1-propenyl) benzene] derived from Foeniculum vulgare Mill—	Reverse phase evaporation technique	PEGylated liposomal *trans*-anethole	Toxicological studies of MCF-7 and T47D cell lines revealed 9- and 8-fold cytotoxicity effects, respectively, when compared to free drugs.	[31]
Breast cancer (MCF-7)	Paclitaxel (PCX)	Film dispersion-extrusion-ammonium sulfate gradient method	PEGylated nanoliposomes, nano-archaeosome, nanoliposomes	Increment in IC_50_ values: PEGylated nanoliposomes < Nanoliposomes < Nano-archaeosomes	[39]
The human breast cancer cell lines SK-BR-3, BT-474 and MDA-MB-231	Paclitaxel and Herceptin (a recombinant humanized monoclonal antibody)	Thin-film hydration method	Paclitaxel-Loaded PEGylated Immunoliposomes	Different activities in: Paclitaxel-Loaded PEGylated Immunoliposomes (PIL) Paclitaxel-Loaded PEGylated liposomes (PL) Paclitaxel-Loaded PEGylated Immunoliposomes (PIL) + Herceptin	[60]
C26 murine colon carcinoma solid tumor model	Rhenium-188	Ready-made PEGylated liposome (Nano-X)	Rhenium-188 in PEGylated nanoliposomes	Increased apoptotic nuclei in^188^Re-liposome-treated mice.	[41]
Breast cancer cells (MCF-7)	Resveratrol, Paclitaxel	Thin-film hydration method	PEGylated liposomes containing Res and/or PTX	The Res liposomes had minimal cytotoxicity, but the PTX liposomes and the composite liposomes indicated cytotoxicity. The cytotoxicity of composite liposomes containing PTX and a greater dosage of Res was the highest.	[62]
SW620 PC-3 MDA-MB-231 A549 U251 U87 HepG2 cell lines	Bufalin	Thin-film evaporation method and high-pressure homogenization method	Bufalin-loaded PEGylated liposomes and Bufalin-loaded liposomes	The cytotoxicity of blank liposomes was determined to be within acceptable limits, but bufalin-loaded PEGylated liposomes were shown to be more hazardous to U251 cells than the bufalin entity. U251 and U87 glioma cancer cells were more susceptible to bufalin than the other cancer cells examined.	[50]
KunMing Mice: Mice ascites tumor H-22 Cells	Copper Oleate liposome Cu(OI)2-L and Disulfiram (DSF)	Alcohol injection method	Copper Oleate Liposome (Cu(OI)2-L) DTC diethyldithiocarbamate	Enhanced tumor inhibition rate with the treatment of Cu(OI)_2_-L and DSF nanoparticles indicating an improved synergistic antitumor effect.	[63]
HT29 colon cancer cells and HEK293T Human embryonic kidney	Curcumin (CUR) and Aptamers (APT)	Nanoprecipitation technique with a slight modification.	Encapsulated CUR (curcumin) with PLGA-lecithin-PEG nanoparticles (CUR-NPs)	–	[70]
Mice xenograft	Gemcitabine (GEM)	Film dispersion-extrusion-ammonium sulfate gradient method	Lipoid S-100, Cholesterol and DSPE-PEG2000 (at a molar ratio of 9:2:0.07)	Intravenous administration showed tumor inhibition rate of GEM-Lip was 6.25-fold as that of GEM-Solution	[71] Scholar
In vitro A172 human and C6 rat glioma cell lines	Carboplatin	Reverse-phase evaporation technique	Lecithin, cholesterol, polyethylene glycol 4000, DSPE-mPEG-2000	Enhanced cyto- toxicity compared with free drug	[72]
The H630WT (passage 13–27) and H630R10 (passage 4–16) cells	Disulfiram	ethanol-based proliposome methods (80–90 nm)	Hydrogenated soya phosphatidylcholine (HSPC) or dipalmitoyl phosphatidylcholine (DPPC) and N-(Carbonyl-methoxypolyethylenglycol-2000)-1,2-distearoyl-*sn*-glycero-3-phosphoethanolamine (DSPE-PEG2000) with cholesterol	Improved stability of disulfiram in serum	[73]
Glioma C6 cells	Doxorubicin and carboplatin	Reverse phase evaporation methods	lecithin, cholesterol, DOX/CB, and DSPE-PEG2000	PEG-Lip-DOX/CB significantly increased the effect of loaded drugs in cytotoxicity by 1.5-fold	[74]

## 5 Results

### 5.1 Search results

**Fig. 3:**
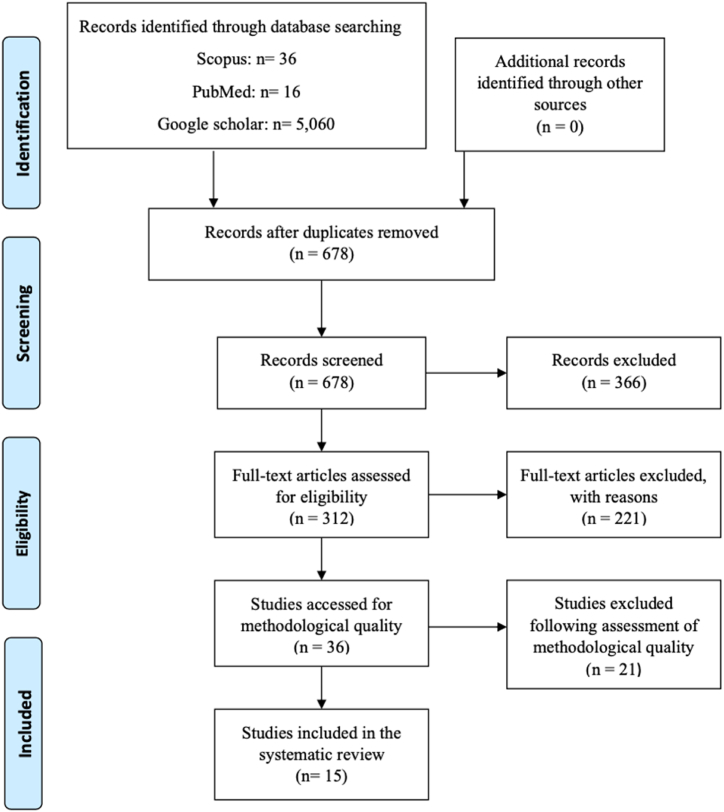
PRISMA diagram of the search method process.

A flow diagram showing the search and selection strategy used in the systematic review is presented in Fig. 3 based on Preferred Reporting Items for Systematic Reviews and Meta-Analyses (PRISMA) method.

Out of 5112 articles, 312 articles remained after screening and removal of duplicates which were strictly done based on the pre-defined inclusion and exclusion criteria. Afterwards, 15 articles met the inclusion criteria from the full-text screening for eligibility while 19 articles were excluded for several reasons, including due to being unfit for systematic review and meta-analysis reporting as referred to in the PRISMA guidelines, lack of reporting data for methodological quality assessment, animal studies, and being unable to be interpreted as well as failure to fulfill the PICO parameters.

### 5.2 Results of the research

The electronic database searches yielded 678 relevant documents using the search term “PEGylated”. Of these 678 documents, 312 were using “PEGylated liposome” AND “cancer”. 71 articles were found “PEGylated liposome” AND “anticancer”. 36 articles were obtained using “PEGylated liposome” AND “anticancer” AND “drug delivery”. The original inquiry yielded a total of 253 titles of possibly relevant articles. The titles and abstracts of 253 documents were reviewed to determine potential relevance, excluding 723 due to irrelevance to the review. We obtained and reviewed 32 full documents and formally excluded 19 (8 had no eligible intervention and 15 used ineligible study designs). A total of 15 studies met all of the eligibility criteria and were included in the review.

## 6 Discussion

Cancer is a serious public health issue that leads to an increase in the number of fatalities globally. Aside from the current high incidence of coronavirus pandemic, cancer has always been a concerning disease across the world. Despite the many treatments that have been provided and are now accessible, including chemotherapy, radiation, and surgery, the mortality rate remains high [31,36]. The effectiveness of today's conventional cancer chemotherapeutics is greatly constrained, resulting in a low therapeutic index and poor patient tolerance. Likewise, the inadequacy of anticancer drugs to reach target areas at therapeutic concentrations due to fast clearance demonstrates the ineffectiveness of the treatment [25,26].

In spite of this, a variety of approaches to coping with pharmaceutical-related issues has been explored. The methods are largely targeted at delivering drugs to only the organs, tissues, or cells that are affected. Liposomal technology is a potential drug delivery method that can encapsulate a huge spectrum of hydrophilic and hydrophobic active compounds while also achieving high target efficiency [23,31]. Furthermore, a variety of functional moieties, including polyethylene glycol (PEG) molecules and targeting ligands, are relatively simple to bind to the liposome surface. PEGylated liposomes are referred to “sterically stabilized liposomes” (S-liposomes), where they have a reduced affinity for the cells of the mononuclear phagocyte system (MPS), making it will be avoid in detection. In addition, it able localize in high concentrations in solid tumors [6].

**Fig. 4:**
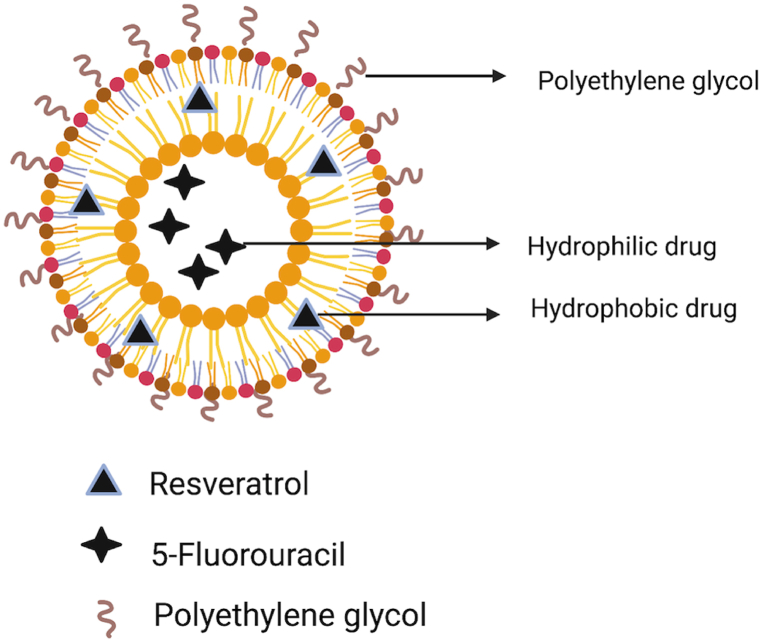
Dual drug-loaded PEGylated liposome diagrammatic representation. Adapted from Ref. [32].

Anticancer drugs are successfully delivered using PEGylated liposomes. The focus of the discussion is on the efficacy of anticancer delivery strategies to suppress cancer cell proliferation as well as the efficiency of single therapy and combination therapy [[29], [37]]. The significant contribution of liposomal anticancer drug delivery is not limited to Doxil® only, it is also used in DaunoXome® (daunorubicin), Depocyet® (cytarabine), Myocet® (cyclophosphamide), Mepact® (mifamurtide), Marqibo® (vincristine) and Onivyde™ (irinotecan) [38]. Fig. 4 illustrates the structure of a PEGylated liposome with 5-FU and resveratrol. The difference between liposome and PEGylated liposome is the additional PEG-chains that are attached to the lipid membrane to enhance drug efficiency.

### 6.1 Effectiveness of PEGylated liposomes in anticancer drug delivery

#### 6.1.1 Single drug therapy

The single therapy using PEGylated liposomes has shown a significant increase in the residence time in the blood circulation as well as in the ability to reach the target sites efficiently. There are developed compounds and natural compounds as the main components of PEGylated liposomes. The main target and function remain the same, and only the production methods are different. Doxorubicin, vincristine, and paclitaxel are examples of the developed compounds frequently used in anticancer therapy. Meanwhile, natural sources of medicinal compounds such as Curcumin and Goniodiol have been recognized for many years and have demonstrated a variety of pharmacological effects including anticancer potential.

##### 6.1.1.1 Anticancer drugs

Paclitaxel is a chosen anticancer drug which has a potent antineoplastic activity against a variety of solid tumors. However, because of its high cytotoxicity, low water solubility, and lipophilic properties, paclitaxel exhibits limited therapeutic applicability. Following this, three distinct anti-tumor therapy methods have been experimented on the MCF-7 cell line.

According to Movahedi, paclitaxel (PCX) is entrapped in liposome, archaeosome, and PEGylated liposome nanocarriers [39]. The Archaebacteria-derived carrier, nanoarchaeosome, is a novel liposomal carrier. In comparison to nanoliposomes and archaeosomes, PEGylated liposomes had the smallest average diameter. Interaction of different charges of PEG with liposomes can become the factor in nanoparticle size reduction. Despite that, all of the three methods indicate high encapsulation efficiency. When compared to liposomes, the PEGylated liposome drug delivery had greater stability and higher loading efficiency, resulting in prolonged drug release. Even though, PEGylated liposomal sirolimus did not enhance the its anticancer activity *in vitro*, but it prolonged the in vivo circulation [40]. The addition of PEG in the formulation improves the drug stability and efficiency in reaching its target sites. In the cytotoxicity assay against MCF-7 cell line, the standard paclitaxel had the highest IC_50_ value followed by liposomes and archaeosomes with PEGylated liposomes showing the lowest IC_50_ value [39].

PEGylated liposomes can also be used in radiotherapy. Chang presented Rhenium-188 as targeted radionuclide therapy and nuclear imaging. A 155 keV gamma photon and a 2.12 MeV beta-particle were emitted [41]. ^188^Re-liposomes also indicate potential therapeutic effectiveness against C26 murine colon cancer solid tumor models, in addition to 5-FU or leucovorin (LV) based chemotherapy. Radiotherapy using PEGylated liposome radionuclide or chemotherapeutic delivery has proved to be effective with the ability to specifically target tumor sites and minimize accumulation [41].

In their work, the scientists conducted two experiments: dose-dependent impact of ^188^Re-liposome treatment on C26 colon tumor-bearing mice and therapeutic efficacy of ^188^Re-liposomes compared to 5-FU in C26 colon tumor-bearing animals. BMEDA, glucoheptonate, acetate solution, stannous chloride, and ^188^Re were combined with PEGylated liposomes in the formulation. As a result, the levels of blood urea nitrogen (BUN), creatinine (CRE), glutamate oxaloacetate transaminase (GOT), and glutamic pyruvic transaminase (GPT) levels became normal, indicating no significant liver or spleen damage. Furthermore, despite the fact that blood cells are more susceptible to radiation than other cell types, the erythrocyte and platelet counts in the mice showed no significant alterations. The primary objective of radiation for cancer treatment is to induce tumor cell death [41].

Meanwhile, the doubling time (DT) of tumor volume and the specific growth rate are used to determine the specific growth rate (SGR). ^188^Re-liposomes increase DT duration and enhances therapeutic efficacy. The average DT in the mice treated with ^188^Re-liposomes rose from 3.84 days to 8.46 days in the C26 colon tumor-bearing mice model. The tumor-bearing mice treated with ^188^Re-liposomes showed a considerably greater mean tumor growth inhibition rate (MGI) and a significantly longer median survival time than the mice treated with the anticancer drug 5-FU or untreated animals. Ultrasound imaging can identify a reduction in the tumor volume and number of blood vessels. Another notable outcome of ^188^Re-liposomes was the average tumor SGR decrease. The improved therapeutic effectiveness of ^188^Re-liposomes against murine colon carcinoma exhibits the potential for future adjuvant oncology application [41]. Exemestane, which were prepared by thin film hydration is poorly soluble and poorly permeable was successfully formulated in a PEGylated nanoliposome [42].

##### 6.1.1.2 Natural compounds

Charoensit et al. demonstrated the efficacy of Goniodiol-loaded PEGylated liposomes against human liver hepatocellular carcinoma cell line (HepG2) [36]. A range of bioactive styryl lactones identified and produced from Goniothalamus species demonstrate a substantial cytotoxic action in both human cancer cells and tumor models. Furthermore, one of the styryl lactones exhibits a cancer-specific cytotoxic effect. The fact that goniodiol has a higher cytotoxic effect on human hepatocellular liver cancer cell lines (HepG2) than on normal mice hepatocytes demonstrates this. As a result, goniodiol has the potential to be promising anticancer therapy for drug-resistant cancer cells while being completely safe for normal cells. In this work, Goniodiol-loaded PEGylated liposomes were made using the thin-film hydration method [36].

The lipophilic portion (benzene and dihydropyrone ring) of Goniodiol inserted into the hydrocarbon chains of the phospholipid bilayer mades the size of Goniodiol-loaded PEGylated liposomes larger than the conventional ones. Even with the addition of amide-linked PEG-DSPE, the encapsulation efficiency remained the same as that of the conventional liposomes [43]. The Goniodiol stability is affected by variations in the liposome membrane permeability. The amide bond length between PEG and lipid molecules has prevented goniodiol leakage by altering the steric effect of PEG molecules [36,43].

Shahbazian formulated *trans*-anethole PEGylated liposomes on MCF-7 and T47D cell lines [31]. The reverse-phase evaporation method was used to prepare the PEGylated liposomes, which can increase the efficiency and reduce the medication adverse effects. Anethole [1-methoxy-4-1(1-propenyl)] is an anti-tumor and anti-inflammatory compound found in fennel (Foeniculum vulgare Mill). The conventional drug and PEGylated liposomal drug were compared in this study. PEGylated liposomal *trans-*anethole showed a larger mean diameter and size distribution, which may reduce the stability of the nanoparticles. PEG, on the other hand, has the ability to enhance drug stability and drug delivery efficiency to the target cells. This is an important aspect to improve treatment effectiveness. As a result, PEGylated liposomal drugs had a higher retention capability and slower release even after 48 h compared to the free form. The *trans*-cytotoxicity of anethole against T47D and MCF-7 cell lines was also particularly noteworthy, with the findings showing eight and nine times higher than the standard drugs [31].

Plumbagin (PLB) is a naturally occurring naphthoquinone that possesses an anticancer activity against various types of cancer cell lines such as melanoma [44], non-small cell lung carcinoma [45], and ovarian cancer [46]. PLB alone exhibits various pharmaceutical issues in conventional cancer chemotherapeutics including low therapeutic index and poor drug solubility. In the study, PLB is found to be more effective for treating melanoma than it is for ovarian cancer.

Plumbagin-loaded PEGylated liposomes were produced by utilizing the thin-film hydration method and evaluated on C57BL/6J mice with B16F1 melanoma [23]. The presence of cholesterol in the formulation can improve the membrane fluidity and provide stability for the bilayer membrane. It was shown by the sustained drug release after the initial burst release phase of PLB over the next 24 h. The lipid bilayer stabilization may become the factor that causes the sustained drug release, and the release of the liposomal surface-bound free drug can be the cause of the burst release. Besides, the steric stabilization effect of the PEGylated liposomes causes unobvious changes in the particle size, and the plasma levels of PLB-loaded PEGylated liposomes remained detectable after 24 h compared to the conventional liposomes and free PLB. PEGylated liposomes were shown to reduce the tumor volume and extend the survival of B16F1 melanoma cells cultured in vivo in C57BL/6J mice. When compared to other free and conventional PLB liposome formulations, the extended circulation of PEGylated liposomes leads to a high local concentration of PLB at tumor sites, indicating an enhanced tumor inhibitory action [23].

Bufalin is a natural broad anticancer spectrum that exhibits cardiotonic, antiviral, and immune regulation. Bufalin is isolated and identified from the toad bufo gargarizans Cantor or *Bufo melanostictus* Schneider's *Venenum Bufonis*, which is the secretion of the skin and parotid venom glands. However, the short half-life, heavy toxicity, as well as low solubility hinder the clinical application of bufalin [47]. Meanwhile, liposomal technology is one of the most promising drug delivery methods, but it has drawbacks such as fast blood clearance and opsonization of plasma proteins [48,49].

Therefore, Yuan made a comparison between bufalin-loaded liposomes and bufalin-loaded PEGylated liposomes [50]. The bufalin-loaded PEGylated liposomes and bufalin-loaded liposomes were prepared through a homogenization-film rehydration method. Compared to bufalin-loaded liposomes, bufalin-loaded PEGylated liposomes had a negative surface charge with a greater absolute value and negative zeta potential, which improve their stability. Moreover, the surface modification shows the slow release of bufalin from the PEGylated liposomes. In this study, bufalin was tested on Male Sprague-Dawley rats with SW620, PC-3, MDA-MB-231, A549, U251, U87, and HepG2 cell lines. The suppression of development in various tumor cells is demonstrated in the cytotoxic study, and it is more sensitive to U251 and U87 glioma cancer cells than to the other cancer cells examined. Bufalin-loaded PEGylated liposomes have been found to prolong the therapeutic window for glioma by increasing blood circulation and prolonging half-life [50]. Stealth liposome of andrographolide was reported in increase cellular internalization of blood brain barrier in artificial membrane [51].

#### 6.1.2 Combination therapy

Apart from single-drug therapy, dual-drug-loaded PEGylated liposomes have demonstrated outstanding results against cancer cells. The idea of utilizing nanoparticles to deliver two drugs at the same time to the diseased site has gained a lot of interest since it allows annihilation of cancer cells by targeting several molecular targets in the cell [[52], [53]]. The successful PEGylated anticancer drug, Doxil® still have some limitation with regard to effectiveness and some severe side effect still limit the use of Doxil® in cancer therapy. Thus, the development strategy by reducing the dose and synergic combination of anticancer drug is urgently needed [9].

**Fig. 5:**
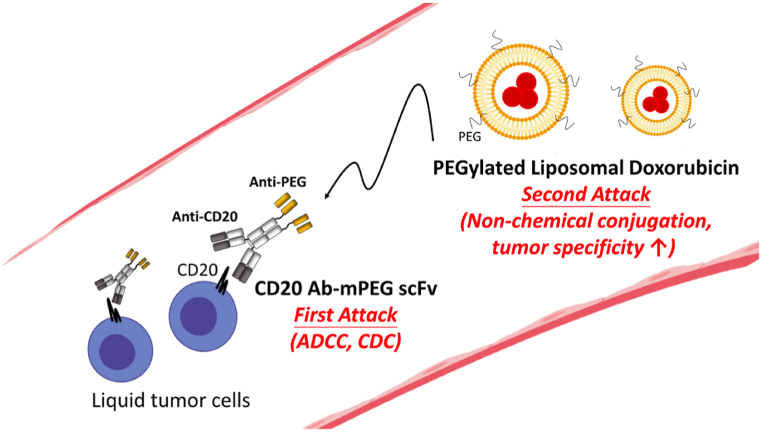
The “double attack” strategy where bispecific antibody combined with PEGylated liposomal doxorubicin (Taken from Ho et al., 2021).

The recent strategy was reported by Ho (2021) reported “double attack “strategy for anticancer using CD20 Ab-mPEG scFv and PEGylated liposomal doxorubicin which incresase cellular internalization of doxorubicin form36.6%–83.6% and enhanced cellular cytotoxicity by 11-fold [54] (Fig. 5).

*Trans*-resveratrol (Res) is a polyphenol and has been proven for its anticancer, cardioprotective, neuroprotective, anti-inflammatory, and anti-diabetic effects. The potential of resveratrol (RES) in chemotherapeutics can outweigh the side effects. However, because of its low bioavailability and rapid clearance, RES is only used as a supplement, not as a potent medication. Therefore, Mohan et al. developed dual drug-loaded PEGylated liposomes containing RES and 5-fluorouracil (5-FU) to eradicate head and neck squamous cell carcinoma cell lines more efficiently [32]. The thin-film hydration technique was used to make the PEGylated liposomes. There was an absence of 5-FU burst release in the dual drug-loaded liposomes in this study, and 5-FU release in the presence of RES was three times slower than in the single drug liposomes [55]. In contrast to 5-FU, which is a polar molecule and does not depend on the drug-to-lipid ratio, RES is a hydrophobic molecule that will impact the encapsulation efficiency depending on the ratio of drug to lipid. As a result, compared to RES, the hydrophilic chains on the liposome surface promote higher trapping of the hydrophilic 5-FU [56,57].

On the other hand, hydrophilic moieties such as polyethylene glycol (PEG) help to circumvent clearance by the reticuloendothelial system and have high colloidal stability, maintaining their size for 24 h in a protein-containing medium [58,59]. According to the median effect principle, a drug combination is slightly antagonistic at GI_50,_ and drug encapsulation lowers the threshold concentration required for synergistic effects when compared to free drug combinations. The liposomal formulation showed changes in cell expression levels. There was downregulation of cells expression levels of *bcl-2*, *cyclin D1*, and *akt* as well as upregulation of *bax* and *caspase-3*, indicating apoptosis. The liposomal carrier causes varied combination effects on gene expression, and the combination of these effects may influence cytotoxicity [32].

Multidrug resistance is also a rising problem in the drug delivery system of cancer treatment. Res and PTX were co-encapsulated in a liposome and used to treat drug-resistant tumors. Apart from improving the in vivo bioavailability, the main advantage of co-encapsulating Res and PTX in the same nanocarrier is the enhanced synergistic effects of the two anticancer therapies. In vitro, composite liposomes show significant cytotoxicity against drug-resistant MCF-7/Adr tumor cells, and they improve drug bioavailability and tumor retention in vivo [60]. MDR is overcome using a composite liposome comprising both PTX and Res. The liposomal formulation resulted in higher blood concentrations, bioavailability, and longer circulation durations. The data suggest that the synergistic impact of Res and PTX may aid in the reversal of drug resistance in cancer cells. The composite liposomes exhibit increased anticancer effects on the drug-sensitive tumor MCF-7 when compared to the PTX liposomes [61]. The composite liposomes have the potential to improve the treatment of both drug-resistant and drug-sensitive malignancies, suggesting that combining Res and PTX has a synergistic anticancer effect on tumor regardless of the MDR status. The findings imply that delivering Res and cytotoxic drugs via a nanocarrier can be useful in the treatment of drug-resistant cancers [62].

Meanwhile, disulfiram (DSF) is a bivalent metal ion chelator that is added into a formulation to improve the Cu^2+^ intracellular transport which is a Cu^2+^ dependent anticancer mechanism. In the research by Zhou, copper-loaded liposomes containing DSF are shown to be effective against H-22 cells [63]. The drawback of Cu^2+^ is probably the potential feature against cancer cells since the combination of DSF and Cu^2+^ exhibits a high ROS production activity against cancer cells but has a slow-release rate. Therefore, the effects of copper oleate solution Cu (OI)_2_–S and copper oleate liposomes Cu (OI)_2_-L on H-22 cells of KunMing mice were compared in this research. The synthesis of copper oleate in this formulation improved the encapsulation efficiency of the PEGylated liposomes while also minimizing the leakage issues that can arise from the use of water-soluble copper to integrate in a liposome [63].

Incorporating a copper chelator-lipid combination in the lipid membrane resulted in a Cu(OI)2-L formulation that was stable in serum and exhibited long-circulating pharmacokinetics in vivo. Cu2+ and DSF may concentrate in malignant cells when Cu (OI)2-L is combined with DSF nanoparticles. The characteristics of Cu (OI)2-L cause drug release in an acidic medium, the vesicles accumulated in tumor tissues, and improve the effectiveness as well as safety while minimizing adverse effects. Through the EPR effect, the tiny vesicle size has the great potential to accumulate in tumor. PEG-modified nanoparticles have the capacity to evade identification by the mononuclear phagocyte system (MPS) [22].

This pH-sensitive PEGylated liposome formulation has the potential to enhance copper release to tumor cells while preventing drug leakage in the bloodstream. Copper liposomes outperform standard oral copper preparations in terms of *in vitro* and in vivo stability as well as the capability to lengthen the circulation period. This can also increase the bioavailability, making it more appropriate to use in combination with disulfiram in anticancer treatment [63].

In an intracranial model of breast cancer using the MDA-MB-231-BR cell line, Anders compared the pharmacokinetics (PK) and efficacy of PEGylated liposomal doxorubicin (PLD) with those of non-liposomal doxorubicin (NonL-doxo) [25]. PLD has a greater pharmacological and efficacy profile than NonL-doxo in an intracranial model of breast cancer brain metastases, resulting in higher and longer plasma and cerebral tumor exposure. In individuals with extracranial advanced breast cancer, PLD also has a more favorable toxicity profile than NonL-doxo [25,64]. Furthermore, when comparing NonLdoxo plus ABT-888 to PARP inhibitor combos in this model, adding the PARP inhibitor ABT-888 to PLD can improve survival [65]. Taken together, the findings point to a novel and promising preclinical treatment for breast cancer brain metastases: liposomal delivery of a chemotherapeutic alone or in combination with a small molecule PARP inhibitor.

The PEGylated technology was also implemented in radioactive materials delivery. The use of PEGylated liposome in gadolinium neutron capture therapy of gadolinium-157 using clinical gadolinium complex (Gadovist®) has improve cancer suppression by 43% compared to control [66].

### 6.2 Challenges in doxorubicin PEGylated liposome

Even though PEGylated liposomes over many advantages compared to conventional liposomes, but some reports mentioned some challenges. PEGylated liposomes were reported to have unexpcetd immunogenic response, rapid clearance after repeated administration [67]. It was proven by increasing of anti-PEG antibody up to 42% in healthy human who are exposed to PEG for many times [68]. With regards to Doxil® itself, it is associated with a wide range toxic effect to the skin due to unique encapsulated form [69].

## 7 Conclusion

This paper has systematically identified fifteen studies of PEGylated liposome strategy on anticancer drug delivery. It showed that PEGylated liposomes in single and combination drug formulation show remarkable results. It is highlighted that PEGylated liposomes exhibit positive effect on prolonging drug circulation, enhancing drug accumulation, permeability, retention and attenuates side effects. Dual drug loading to liposomes promises an enhancement of drug efficacy. Therefore, the PEGylated liposomes have a higher opportunity to be more effective anticancer treatment in the future. The use of PEGylated liposomal delivery might be extended to infectious disease caused by bacteria, fungal and virus. However, the effectiveness of PEGylated liposome in the delivery of drugs for certain target should be thoroughly investigated to achieve the optimum and safe effect.

## Author contribution statement

Muhammad Taher: Conceived and designed the experiments; Performed the experiments; Analyzed and interpreted the data; Wrote the paper.Aina Atiqah Rushdan: Conceived and designed the experiments; Performed the experiments; Wrote the paper. Deny Susanti: Conceived and designed the experiments; Performed the experiments; Analyzed and interpreted the data.Muhammad Salahuddin Haris, Riyanto Teguh Widodo, Yandi Syukri, Junaidi Khotib: Analyzed and interpreted the data.

## Funding statement

This work was supported by the Ministry of Education, Culture, Research and Technology of the Republic of Indonesia via Research Grant PDUPT.

### Data availability statement

No data was used for the research described in the article.

## Declaration of competing interest

The authors declare that they have no known competing financial interests or personal relationships that could have appeared to influence the work reported in this paper.
